# Efficient traceless modification of the P1 bacteriophage genome through homologous recombination with enrichment in double recombinants: A new perspective on the functional annotation of uncharacterized phage genes

**DOI:** 10.3389/fmicb.2023.1135870

**Published:** 2023-03-20

**Authors:** Agnieszka Bednarek, Katarzyna Giermasińska-Buczek, Małgorzata Łobocka

**Affiliations:** Institute of Biochemistry and Biophysics of the Polish Academy of Sciences, Warsaw, Poland

**Keywords:** bacteriophage P1, temperate bacteriophage, prophage, bacteriophage engineering, targeted mutagenesis, homologous recombination, multicopy plasmid, ampicillin resistance

## Abstract

The advent of high-throughput omic technologies has caused unprecedented progress in research on bacteriophages, the most abundant and still the least explored entities on earth. Despite the growing number of phage genomes sequenced and the rejuvenation of interest in phage therapy, the progress in the functional analysis of phage genes is slow. Simple and efficient techniques of phage genome targeted mutagenesis that would allow one to knock out particular genes precisely without polar effects in order to study the effect of these knock-outs on phage functions are lacking. Even in the case of model phages, the functions of approximately half of their genes are unknown. P1 is an enterobacterial temperate myophage of clinical significance, which lysogenizes cells as a plasmid. It has a long history of studies, serves as a model in basic research, is a gene transfer vector, and is a source of genetic tools. Its gene products have structural homologs in several other phages. In this perspective article, we describe a simple and efficient procedure of traceless P1 genome modification that could also serve to acquire targeted mutations in the genomes of certain other temperate phages and speed up functional annotations of phage genes.

## Introduction

Bacteriophage P1 is a model temperate tailed myovirus of known genomic sequence (93.6 kb) isolated from *Escherichia coli* (Bertani, 1951; Yarmolinsky and Sternberg, 1988; Łobocka et al., 2004). It can develop lytically or lysogenize representatives of different genera of the *Enterobacteriaceae* and *Rhizobiaceae* families and can serve as a DNA donor to certain infection-proficient bacteria even if they cannot support its propagation (Kaiser and Dworkin, 1975; Murooka and Harada, 1979; O'Connor and Zusman, 1983; Yarmolinsky and Sternberg, 1988; Giermasińska and Łobocka, 2016; Keller et al., 2021). In lysogens, P1 is maintained as a unit-copy circular plasmid. P1-related prophages or plasmids are prevalent in natural isolates of *E. coli, Klebsiella, Salmonella*, and *Shigella* (Gilcrease and Casjens, 2018; summarized by Łobocka and Gagała, 2021). Some of these prophages and plasmids undergo frequent transmission among bacteria of human gut microbiota or carry antibiotic resistance determinants, making P1 a phage of clinical significance (Colomer-Lluch et al., 2011; Billard-Pomares et al., 2014; Bai et al., 2017; Yang et al., 2017; Pfeifer et al., 2022). Owing to its transducing potential, wide host range, and the ability to form lysogens, P1 has played an important role in the genetic mapping of *E. coli* chromosomes, in studies on basic molecular processes, and acquisition or construction of genetic engineering tools (Lennox, 1955; Tyler and Goldberg, 1976; Singer et al., 1989; Schofield et al., 2001; Westwater et al., 2002; Lehnherr, 2006; Wachsman and Heidstra, 2010; Huang and Masters, 2014; Yarmolinsky and Hoess, 2015). It is still among the most commonly used tools for general transduction in bacterial genome engineering (Thomason et al., 2007).

Early studies on P1 allowed mapping of certain mutations abolishing or modifying P1 development, morphology, or plasmid maintenance functions (summarized in Yarmolinsky and Sternberg, 1988 and in Łobocka et al., 2004). However, despite the determination of a complete P1 genomic sequence and identification of major P1 virion components (Łobocka et al., 2004; Gonzales et al., 2022), the functions of several P1 genes are unknown. Meanwhile, the ability of P1 to lysogenize cells provides a possibility to functionally analyze the P1 genome by knocking out or modifying its genes one by one through targeted mutagenesis, even if the constructed mutants could not be propagated as phages. The progress in such analysis depends on the techniques of mutant construction and the efficiency of mutant recovery. The results should not only help in the functional assignment of P1 proteins of unknown roles but also provide hints as to the functions of similar proteins encoded by phages, which cannot be easily studied.

## Selection of double recombinants to acquire P1 mutants without using markers selective for the mutations

The introduction of any mutation to prophage DNA by homologous recombination between the prophage and donor DNA with a mutation, cloned in a plasmid, requires double crossover between homologous regions flanking the target and the mutant fragments. While a single crossover leads to plasmid integration with phage DNA, the second crossover leads to plasmid excision, which eventually results in the exchange of the wild-type and mutant fragments. In the method described here, we took advantage of the possibility of separating these reactions. In addition, we found a way to distinguish lysogens with single prophage recombinants from those in which the plasmid was excised from the prophage. Using the latter for prophage induction appeared to be a simple strategy to significantly increase the frequency of desired mutant recovery, so there is no need to use any selective marker in donor DNA.

**Figure 1:**
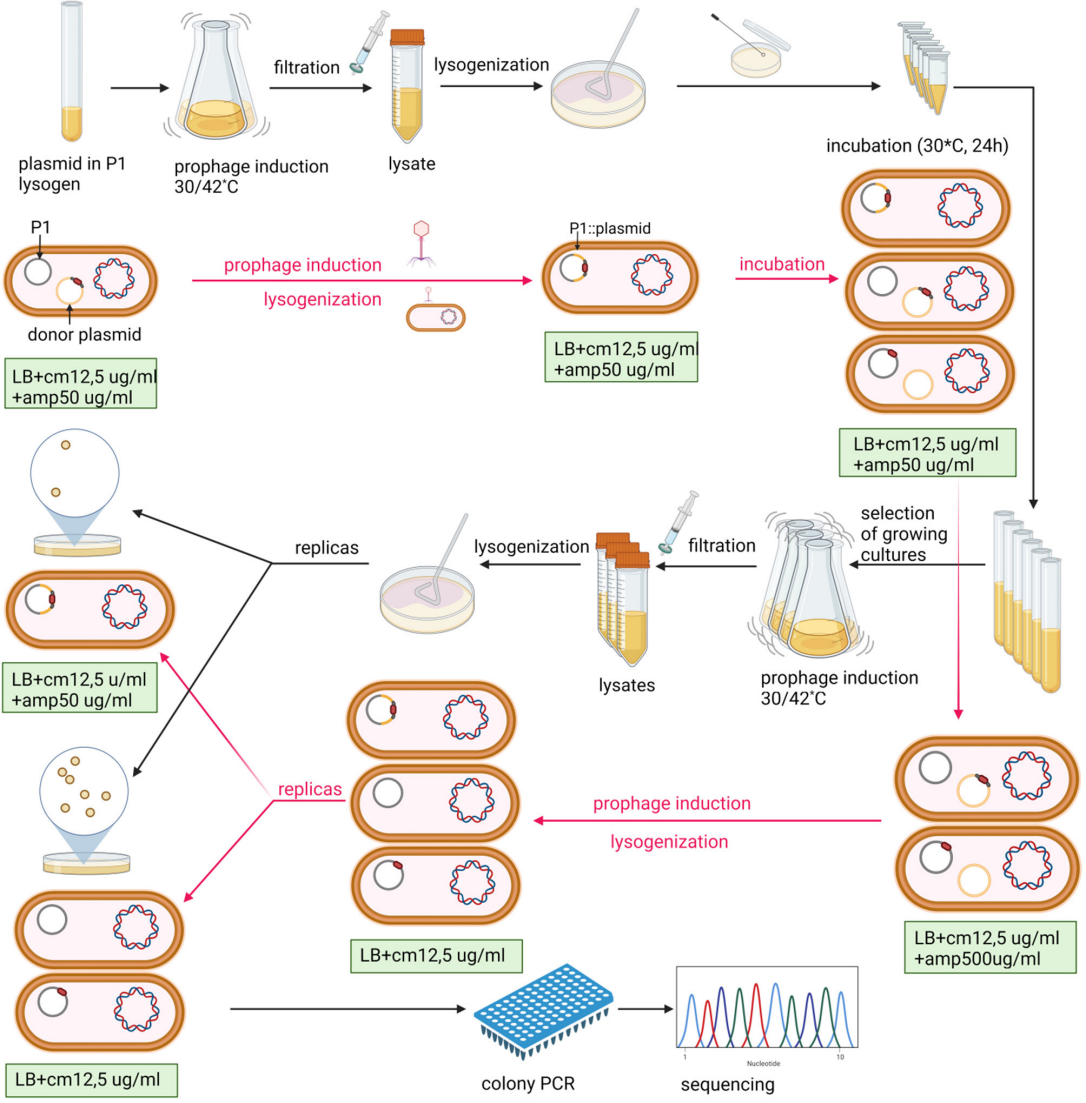
Scheme of P1-targeted mutation acquisition by homologous recombination and enrichment of recombinant pool in double recombinants. The P1 designation in the figure indicates the P1 *c1-100 mod749::*IS*5* IS*1::*Tn*9* mutant. *E. coli* strain N99 (*galK2*, str^R^) (Gottesman and Yarmolinsky, 1968) was used as a host for the P1 prophage in all experiments. Black arrows indicate the order of laboratory procedures and red arrows indicate the revevant changes in the content of cells at various stages of the experiment. Created with BioRender.com.

In our attempts to construct targeted P1 mutants by homologous recombination using the P1 DNA fragments with mutations, cloned in high-copy number plasmids with an ampicillin resistance determinant, we observed that while P1 lysogens containing a given plasmid with a P1 DNA insert (e.g., pUCP1/x or pBRP1/x, where x is the insert designation) in a free form are resistant to ampicillin at high concentrations (≥500 μg/ml), lysogens containing such plasmid integrated with the P1 prophage are sensitive to ampicillin at high concentrations while remaining resistant to ampicillin at low concentrations. We used this difference in the sensitivity to ampicillin to enrich the population of P1 bacteriophages obtained by the induction of thermosensitive lysogens containing different pUCP1/x or pBRP1/x donor plasmids with the progeny of prophages in which a given donor plasmid was initially inserted in the P1 genome as a result of single homologous recombination but recombined out (as a result of second homologous recombination). This strategy significantly increased the recovery of mutations introduced to P1 progeny by the recombination between the P1 prophage and the P1 DNA fragment with a mutation cloned in a plasmid (Figure 1, Table 1 and Supplementary Table S1). In brief, the P1 bacteriophage obtained by the thermal induction of P1 *mod749::*IS*5 c1-100* IS*1*::Tn*9* (cm^R^) lysogen, as described previously (Bednarek et al., 2022), was used to infect cells of *E. coli* containing a pUCP1/x or pBRP1/x plasmid with a desired mutation in the cloned fragment of P1 DNA. Lysogens selected on LB medium with chloramphenicol (12.5 μg/ml, selective for the incoming phage) and ampicillin (100 μg/ml; selective for the resident plasmid) were purified on the same medium. Next, they were used to inoculate liquid medium with a lower ampicillin concentration (50 μg/ml; selective for the integrated as well as free plasmid), and served to induce a prophage. The phages obtained were used to lysogenize *E. coli* cells. To obtain only lysogens of phages that contained a desired plasmid integrated with phage DNA, the lysogens were selected on LB solid medium supplemented with chloramphenicol (12,5 μg/ml) and ampicillin (50 μg/ml). In total, 8–10 single colonies of lysogens were used to inoculate the LB medium (1 ml) with chloramphenicol (12.5 μg/ml) and ampicillin (50 μg/ml). The cultures were grown overnight with shaking at 30°C to increase the chance of recombination and transferred to fresh portions of LB medium (10 ml) with chloramphenicol (12.5 μg/ml) and ampicillin (500 μg/ml) permissive only for the growth of those cells of lysogens in which the integrated plasmid recombined out from the prophage genome. Cultures of lysogens that increased their optical density in this medium after overnight incubation were used for the thermal induction of the prophage. The obtained phages were used to lysogenize *E. coli* cells. Lysogens were selected on LB solid medium supplemented only with chloramphenicol (12.5 μg/ml) and screened for the ability to grow on a medium supplemented with ampicillin (50 μg/ml) to eliminate those that contained the plasmid inserted in the prophage genome. Lysogens that were sensitive to ampicillin were screened for the presence of the desired mutation in the P1 genome upon thermal prophage induction by restriction digestion or sequencing of the relevant P1 genome region amplicon.

**Table 1:** Influence of the P1 population enrichment in double recombinants on the frequency of recovery P1 mutants created by homologous recombination between prophage P1 and donor DNA in a plasmid.

**Experi-ment**	**Resident *P1 c1-100 mod749::IS5 IS1::Tn9* mutation^a^**	**Introduced mutation^e^**	**Flanking regions of homology in bp (L/R)^b^**	**Number of P1 mutants recovered without enrichment^c^**	**Number of P1 mutants recovered upon enrichment**
1	Unchanged	*lydD*::kan^R^	221/648	1/100	36/100
2	Unchanged	*pdcB*::kan^R^	367/443	0/100	7/100
3	Unchanged	*lydC*Δ12_13TG	434/1628	0/100	12/100
4	*lydCΔ12_13TG*	*lydB*Δ18_420	603/272	NT	2/34
5	Unchanged	*lydA::195_196GATC*	239/1526	NT	2/13
6	*Δlyz::R_λ_*	*lydA::195_196GATC*	239/1526	NT	3/24
7	Unchanged	*lydD*Δ16_81	456/399	NT	1/80
8^d^	Unchanged	*parAB*::kan^R^	491/388	0/100	0/100

a The P1 c1-100 mod749::IS5 IS1::Tn9 bacteriophage and its mutants used in this study to introduce additional mutations were the same as described in Bednarek et al. (2022).

b L/R—left/right.

c NT—not tested.

d No ampicillin-sensitive colonies of this mutant lysogen could be recovered upon lysogenization of *E. coli* with phages obtained upon induction of P1 lysogens grown in the presence of ampicillin at a high concentration.

e The functional assignments of genes targeted in this study are the following: pinholin (*lydD*), unknown (*pdcB*), holin (*lydC, lydA*), antiholin (*lydB*), and plasmid partition (*parAB*).

## Efficiency of mutant recovery from the phage progeny population enriched in double recombinants

As a proof of the concept, when the aforementioned enrichment method was used to insert the kanamycin resistance cassette to the P1 *lydD* or *pdcB* gene, but without the use of kanamycin for mutant selection, the efficiency of desired mutant recovery was up to nearly 40 times higher than that with the use of a similar procedure but without the enrichment step (Table 1, Exp. 1). Consistently, various insertion and deletion mutations (indels) in P1 genes were recovered with such high efficiency that the screening of 10–30 lysogens obtained upon the enrichment step was in most cases sufficient to recover the desired mutant (Table 1, Exp. 3–6). Moreover, the efficiency of mutant recovery seemed to correlate with the number of cultures grown upon transfer to medium with high ampicillin concentration, indicative for plasmid excision from the prophage. While typically cultures of most of the 10 lysogens transferred to this medium grew, in the case of experiment 7, when we recovered only one mutant per 80 tested clones, only three cultures grew upon transfer (Table 1, Exp. 7). Our procedure appeared to be unsuitable only for the recovery of P1 mutant with the insertion of a kanamycin resistance cassette inactivating the P1 partition operon *parAB*, which is essential for the stable maintenance of P1 plasmid prophage (Table 1, Exp. 8) (Austin and Abeles, 1983). Prophages that acquired the mutation upon plasmid recombining out were in most cases immediately lost from cells, when kanamycin was not used for their selection. Consistently, we did not have problems with their recovery when we used kanamycin for their selection.

## Discussion and future perspectives

The development of simple methods of bacteriophage genome engineering is a necessary step in studies on the functions of phage genes and the construction of phages with new properties. Several methods of phage-targeted mutagenesis have been implemented, but even in the case of temperate phages, they have limitations associated with them, e.g., low efficiency, the requirement of specific genetic tools or sophisticated methodology, applicability only to a single phage-host system, or the limited type of mutations that can be introduced (Chen et al., 2019; Łobocka et al., 2021). The method presented here is simple, efficient, does not require any specific genetic tools, and allows the introduction of various kinds of changes to phage DNA tracelessly, without the need to use a selective marker for the introduced change. Like the previous methods described, it is based on RecA-mediated homologous recombination between phage DNA and donor DNA cloned in a plasmid between sequences homologous to the flanks of a target phage gene (Chen et al., 2019). In this system, the acquisition of mutation by the phage requires plasmid insertion and plasmid excision from phage DNA. As a result, some double phage recombinants acquire the mutation. They can form a progeny if the mutation does not abolish the function of the essential phage gene. However, the progeny of phages released from cells used as a recombination platform contains single and double recombinants, as well as phages that did not participate in any recombination event. Thus, in examples described previously, the desired mutants were recovered with low efficiency (10^−7^-10^−3^), and their detection required plaque hybridization with specific probes, which is too tedious and too expensive to study a large number of genes (Sarkis et al., 1995; Oda et al., 2004; Tanji et al., 2004; Namura et al., 2008). Here, we show, using temperate phage P1 as an example, that applying a high-copy number plasmid carrying the ampicillin resistance marker as a vector for donor DNA enables selection from among cells that served as a recombination platform, preferentially the cells with double-phage recombinants. Our procedure appeared to be so efficient in the enrichment of the phage population with double recombinants that screening of 10–30 lysogens obtained upon the enrichment was in most cases sufficient to recover a desired mutant. A limitation of our method is that its success depends on the lytic propagation of mutant phages. Thus, one cannot use it to isolate lack-of-function mutants in essential genes unless the cells used for the lysogenization in the last step contain a relevant plasmid providing the complementing function. However, mutants with slight modifications of certain essential proteins, e.g., extending the recognition specificity of tail fibers, should be possible to isolate.

Two methods were used previously for the targeted mutagenesis of P1: the bacteriophage recombineering with electroporated DNA method (BRED) and the Datsenko and Wanner method (Datsenko and Wanner, 2000; Fehér et al., 2012; Murphy, 2016; Piya et al., 2017; Gonzales et al., 2022). Both of them are based on homologous recombination between the P1 genome and electroporated linear donor DNA with short (~50 bp) regions of homology to flanks of the target P1 gene. The recombination function and protection of donor DNA ends from degradation are provided by the λ Red system proteins (Gam, Bet, and Exo), the genes of which are expressed from a plasmid. In the BRED method, donor DNA and P1 phage DNA are electroporated together into Red-producing cells, and the recombinants carrying the desired mutation are recovered by the screening of plaques (Fehér et al., 2012). The P1 mutant deprived of the IS*1* insertion sequence was constructed in this way, but the method required tedious optimization and suffered from the low efficiency of 94-kb P1 DNA electroporation to cells (Fehér et al., 2012). The method of Datsenko et al. was used to replace certain P1 genes with the kanamycin resistance cassette (Piya et al., 2017; Gonzales et al., 2022). Lysogens of P1, carrying a Red-expressing plasmid, were electroporated with linear donor DNA and used for the induction of P1 lytic development. The obtained phages were used to lysogenize cells, but a selective marker in the donor DNA (the kanamycin resistance gene) was required to select the lysogens of mutants. It could be removed in cells expressing the yeast FLP recombinase gene from a plasmid if the marker was flanked by FRT sites—FLP recognition targets (Datsenko and Wanner, 2000). However, the removal requires an intracellular source of FLP recombinase and always retains an 82–85-bp scar in place of the disrupted gene, which may cause polar effects and limit the use of this method to knockout the last genes in operons. In our method, the mutation of interest in donor DNA needs to be flanked by longer fragments of homology to a prophage compared with the BRED method and the Datsenko and Warner method to satisfy the requirement of RecA-mediated recombination (Table 1). However, our method outperforms both of the aforementioned methods in several other aspects. It is fast and simple. It can be used to introduce subtle changes to phage DNA, such as nucleotide substitutions and small and larger insertions or deletions. It does not require any selective marker in the donor DNA or any extra plasmids providing recombination functions. In addition, it does not require tedious optimization and can serve to modify a phage genome of any size.

In studies presented here, the acquisition of mutations in a few genes of P1 served as an example of our method utility. In further perspective, numerous other genes of P1 could be mutated that way to elucidate their roles or to obtain P1 derivatives of new properties. Our method can be potentially used also for the modification of genomes of certain other phages. Its limitation is the applicability only to temperate phages, the hosts of which are sensitive to ampicillin and can be transformed with high-copy number plasmids carrying an ampicillin resistance marker. The criteria of suitable hosts are met not only by *E. coli* but by at least some other Gram-negative bacteria, such as *Shigella, Salmonella, Klebsiella*, and *Yersinia*. Several their strains could be transformed with the pBR322 plasmid or its derivatives carrying an ampicillin resistance marker (Gómez-Eichelmann, 1979; Corton et al., 1987; Bukholm et al., 1990; O'Callaghan and Charbit, 1990; Shireen et al., 1990; Trevors, 1990; Binotto et al., 1991; Roy et al., 1995). The use of our method to acquire and study mutants of certain temperate phages that infect these bacteria could significantly expand the list of functionally annotated phage genes.

## Data availability statement

The original contributions presented in the study are included in the article/Supplementary material, further inquiries can be directed to the corresponding author.

## Author contributions

AB developed the concept of studies, performed a major part of the experiments, wrote the manuscript draft, prepared the artwork, and provided funding. KG-B performed part of the experiments. MŁ developed the concept of studies, wrote the manuscript draft and final version of the manuscript, and provided funding. All authors approved the manuscript.
