# Supplementary material for: Efficient traceless modification of the P1 bacteriophage genome through homologous recombination with enrichment in double recombinants: A new perspective on the functional annotation of uncharacterized phage genes
Source: Front Microbiol. 2023 Mar 20;14:1135870. doi: 10.3389/fmicb.2023.1135870 (PMC10067587; doi:10.3389/fmicb.2023.1135870)
Supplement: Supplementary file 1 [file Table_1.pdf]

Table S1. Plasmids used as donors of P1 DNA fragments with mutations

Plasmid	Cloned genes or genome fragments	Description (comments)[a,b]	Replication origin /Selective marker	Source or reference
pAKI1	*trpPO lyz*$^+$ *lydD::kan*^{R} *lydE*$^+$	A derivative of pBR322 plasmid containing the cloned P1 *lyz* operon with the *lydD* gene inactivated by the insertion of kanamycin resistance cassette.	pMB1/Amp^R, Kan^R	Bednarek et al., 2022
pAKI2	*cin*$^+$ *lydCΔ12_13TG lydA'*	A derivative of pUC18 plasmid containing a cloned fragment of P1 DNA with the *lydC* gene inactivated by the deletion of two nucleotides at pos. 12 and 13 of *lydC* gene.	pMB1/Amp^R	Bednarek et al., 2022
pAKI3	*lydC*$^+$*lydA::195_196GATC hdf*$^+$ *darA'*	A derivative of pUC18 plasmid containing a cloned fragment of P1 DNA with the *lydA* gene inactivated by the insertion of four nucleotides at pos. 195-196 of *lydA*.	pMB1/Amp^R	Bednarek et al., 2022
pAKI25	*lydBΔ18_420*	A derivative of pPGBT30-*tacPOkorB* plasmid containing a cloned fragment of P1 DNA in which the *lydB* gene was inactivated by the deletion of nucleotides from pos. 18 to 420 of *lydB*. Constructed by the replacement of SacI-SalI fragment with the P1 genome fragment (pos. 29845-30434) containing the fragment of *hdf* gene obtained by amplification with primers oAKI51 (5'TATAGAGCTCTTGCTTAAGACGACGTTTGAG) and oAKI57 (5'TATAGTCGACGATGTGTCAGATGACGAAAAATAAGTA) and digested with SacI and SalI, and the replacement of SalI-MfeI fragment with the P1 genome fragment (pos. 30842-31114) containing the fragment of *lydA* gene, obtained by amplification with primers oAKI54 (5'-TATACAATTGCGTTCCTGATGGATGTCCGAG) and oAKI58 (5'-TATAGTCGACACCCCAGCCAATCATTTCCC), and digested with MfeI and SalI.	pMB1/Amp^R	This work
pAKI26	*lyz*, *lydDΔ16-81*, *lydE*	A derivative of pUC18 plasmid containing a cloned fragment of P1 DNA with the *lydD* gene inactivated by the deletion of nucleotides from pos. 16 to 81 of *lydD*.	pMB1/Amp^R	Bednarek et al., 2022
pUCP1/61	'*pdcA*, *pdcB*, *lpa*'	pUC18-based plasmid containing a fragment of P1 genome library (from pos. 88662 +/-10 to 90086 +/-10 of P1 *mod749*::IS*5 c1-100* genome), blunt-ended and inserted into HincII site.	pMB1/Amp^R	Łobocka et al., 2004
pKGI2	'*pdcA*, *pdcB*::kan^R, *lpa*'	A derivative of pUCP1/61 in which the NcoI-PflMI fragment of *pdcB* gene was replaced by the NcoI-PflMI fragment of amplicon obtained with pUC4K plasmid as a template and primers: OMLO613 (5'-ATCCATGGGCGCTGAGGTCTGCCTCGTGAAGA) and OMLO614 (5'-ATTCCATGGAAAGCCACGTTGTGTCTCAAAATC).	pMB1/Amp^R, Kan^R	This work
pKGI3	*parAB*::kan^R	A derivative of pBEF116 carrying the kanamycin resistance cassette of pUC4K in place of XhoI-MluI fragment containing the C-terminal moiety of P1 *parA* and the N-terminal moiety of *parB* (corresponding to pos. 58544-61051 in the genome of P1); constructed by the insertion in place of XhoI-MluI fragment of pBEF116, of XhoI and MluI-digested amplicon obtained with pUC4K as a template and primers OMLO622 (5'-ATCTCGAGGCGCTGAGGTCTGCCTCGTGAAGA) and OMLO623 (5'-ATTACGCGTAAAGCCACGTTGTGTCTCAAAATC).	pMB1/Amp^R, Kan^R	This work

[a]Positions of sequences derived from the P1 bacteriophage genome refer to the sequence deposited in GenBank under the acc. number AF234172.1. Fragments of primer sequences encoding the restriction sites are underlined
[b]The pGBT30-*tacPOkorB*, pUC4K and pBEF116 plasmids used for the construction of indicated donor plasmids were described previously (Jagura-Burdzy et al., 1991; Taylor and Rose, 1988, Łobocka and Yarmolinsky, 1996, respectively)

Supplementary References

Jagura-Burdzy, G., Macartney, D. P., Zatyka, M., Cunliffe, L., Cooke, D., Huggins, C., et al. (1999). Repression at a distance by the global regulator KorB of promiscuous IncP plasmids. Mol. Microbiol. 32, 519–532. doi: 10.1046/j.1365-2958.1999.01365.x

Łobocka, M., Yarmolinsky, M. (1996). P1 plasmid partition: a mutational analysis of ParB. J. Mol. Biol. 259, 366–382. doi: 10.1006/jmbi.1996.0326

Taylor, L. A., and Rose, R. E. (1988). A correction in the nucleotide sequence of the Tn903 kanamycin resistance determinant in PUC4K. Nucl. Acids Res. 16, 358–358. doi: 10.1093/nar/16.1.358
